# Morphological Characterization, Variability and Diversity among Vegetable Soybean (*Glycine max* L.) Genotypes

**DOI:** 10.3390/plants10040671

**Published:** 2021-03-31

**Authors:** Nagaraju Shilpashree, Sarojinikunjamma Nirmala Devi, Dalasanuru Chandregowda Manjunathagowda, Anjanappa Muddappa, Shaimaa A. M. Abdelmohsen, Nissren Tamam, Hosam O. Elansary, Tarek K. Zin El-Abedin, Ashraf M. M. Abdelbacki, Veerabhadregowda Janhavi

**Affiliations:** 1 College of Horticulture, Vellanikkara, Kerala Agricultural University, Thrissur 680654, Kerala, India; shillushilpanrs999@gmail.com (N.S.); nirmaladevi.sarojini@gmail.com (S.N.D.); 2 College of Horticulture, University of Horticultural Sciences, Karnataka 560065, Bengaluru, India; m_anjanappa@rediffmail.com; 3 ICAR-Directorates of Onion and Garlic Research, Rajgurunagar, Pune 410505, Maharashtra, India; 4 Department of Physics, Faculty of Science, Princess Nourah Bint Abdulrahman University, Riyadh 84428, Saudi Arabia; shamohamed@pnu.edu.sa (S.A.M.A.); nmtamam@pnu.edu.sa (N.T.); 5 Plant Production Department, College of Food and Agricultural Sciences, King Saud University, P.O. Box 2455, Riyadh 11451, Saudi Arabia; 6 Floriculture, Ornamental Horticulture, and Garden Design Department, Faculty of Agriculture (El-Shatby), Alexandria University, Alexandria 21545, Egypt; 7 Department of Geography, Environmental Management, and Energy Studies, APK Campus, University of Johannesburg, Johannesburg 2006, South Africa; 8 Department of Agriculture & Biosystems Engineering, Faculty of Agriculture (El-Shatby), Alexandria University, Alexandria 21545, Egypt; drtkz60@gmail.com; 9 Department of Plant Pathology, Faculty of Agriculture, Cairo University, Cairo 12613, Egypt; amaeg@hotmail.com; 10 Department of Physics, Bengaluru North University, Tamaka, Kolar 563103, Karnataka, India; janu.jj9@gmail.com

**Keywords:** cluster, genetic advance, genotypic variation, heritability, phenotypic variation

## Abstract

Vegetable soybean production is dependent on the development of vegetable type varieties that would be achieved by the use of germplasm to evolve new agronomically superior yielding vegetable type with beneficial biochemical traits. This can be accomplished by a better understanding of genetics, which is why the research was conducted to reveal the quantitative genetics of vegetable soybean genotypes. Genetic variability of main morphological traits in vegetable soybean genotypes and their divergence was estimated, as a result of the magnitude of genotypic variation (GV), and phenotypic variation (PV) of traits varied among the genotypes. All traits showed high heritability (*h*^2^) associated with high genetic advance percentage mean (GAM). Therefore, these variable traits are potential for genetic improvement of vegetable type soybean. Genetic diversity is the prime need for breeding, and the magnitude of genetic diversity values were maximized among specific genotypes. Eight clusters were found for all genotypes; cluster VIII and cluster I were considered to have the most diversity. Cluster VIII consisted of two genotypes (GM-6 and GM-27), based on the mean outcomes of the high yield attributing traits. Hence, these two (GM-6, GM-27) genotypes can be advanced for commercial cultivation; furthermore, other genotypes can be used as source of breeding lines for genetic improvement of vegetable soybean.

## 1. Introduction

Vegetable soybean has been botanically known as *Glycine max* (L.) Merrill (2n = 40) belonging to the family Fabaceae. It is considered an improved vegetable crop over pulse crop soybean, and an immature soybean pod consumed as a vegetable or a snack. In North America, it has been known as green soybean, or edible soybean, Edamame in Japan and Maodou in China. In China, USA, Taiwan and Japan, and it is widely grown as a source of nutrients and calories in vegetable cuisine [1,2], hence it became an important cash crop [3]. Vegetable soybean has been harvested for immature pod at R6 (green pod with full green seeds) stage [4]. It has wider acceptance in Japan and China [5], as a rich source of proteins, vitamin A, C and E, unsaturated fats, thiamine and riboflavin, mainly due to consumer’s preference regarding its characteristic pleasing aroma and sweet pod taste. Vegetable soybean has several medicinal components including lactose free fatty acids, vitamins (D, K, C, folic acid, nicotinic acid, thiamine, riboflavin, pantothenic acid, pyridoxineand biotin, and isoflavones including genistein [6].

The vegetable soybean genotypes are of short duration (65–75 days crop), as a result, fast crop rotation was feasible, resulting in a high yield of approximately 40 tons per hectare [7]. Although vegetable soybeans aren’t commonly used at the moment, they have a lot of potential for widespread cultivation in the future, and identification of suitable vegetable types will help to improve the nutritional security of human beings. Hence efforts were made to identify the high yielding vegetable soybean varieties for commercial cultivation. In order to develop the appropriate vegetable soybean from the pulse soybean, research group involvement is needed in this crop.

The genetic enhancement of most agricultural crops is largely dependent on available genetic variability and diversity, important variation was observed in nine characteristics, including phenotypic coefficient of variation (PCV) and maximum genotypic coefficient of variation (GCV) for pods per plant and plant height, among the 24 soybean genotypes [8].The fact that PCV is higher than GCV means that the climate affected the expression of the traits. The discovery of high heritability and a discernible genetic progress could help in soybean improvement selection [8]. The 61 genotypes found major differences for 15 traits, with higher PCV than GCV, but the depicted variations were too similar for all traits, indicating that environmental influences had no effect on trait expression. The presence of high GCV, PCV, heritability and GA could indicate the likelihood of trait selection based on genotypic variation [9]. The 40 soybean genotypes were identified by agro-morphological grouping (A and B). Cluster A contained 80% of the total genotypes (32) with high yielding traits, while cluster B had 15% of the genotypes (6). It was concluded that cluster A could be used for direct breeding of high yielding lines [10].

It is difficult to assess the effect of variability due to heritability or environmental factors. This could be explained by the effects of heritable and non-heritable elements affecting the total variability. As a result of heterogeneity, breeder could pick up the right genotypes from variable populations that inherit through progenies. In this regard, an analysis was carried out to classify genotypes in order to assess heritability, genetic heterogeneity, genetic advance percentage mean and divergence present among the genotypes of vegetable soybean. As a result, trait estimates may aid in the identification of suitable genotypes, as well as assisting breeders in the selection of diverse parents for breeding and the adoption of effective breeding methodologies, which may aid in the genetic improvement of vegetable soybeans.

## 2. Results

**Figure 1:**
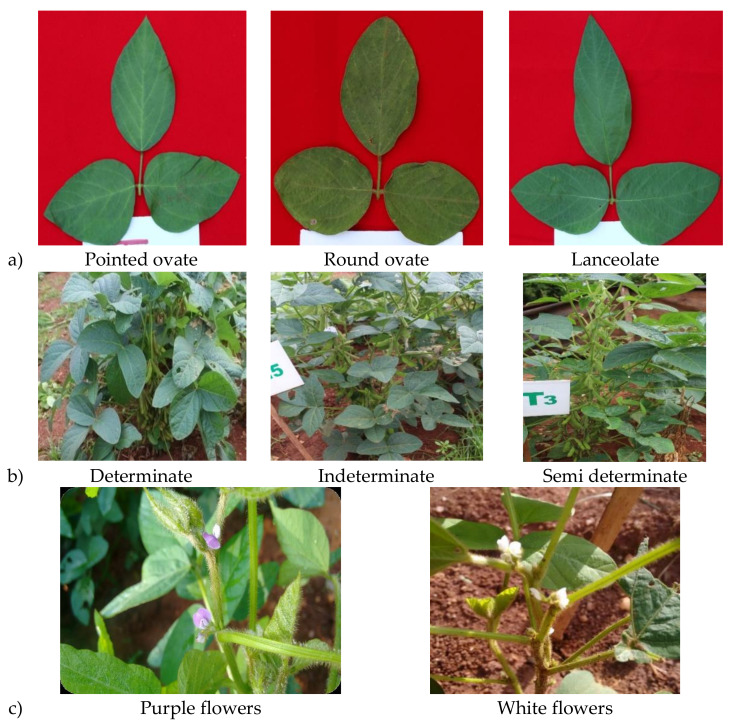
Morphological variabilities among the genotypes of vegetable soybean, (**a**) variability in leaf shape, (**b**) variability in plant habit and (**c**) variability in flower color.

The soybean descriptors of International Board for Plant Genetic Resource (IBPGR), Rome [11], were used to classify the 28 genotypes of vegetable soybean. The morphological characters namely leaf shape, leaf color, flower color, growth habit (Figure 1), color, pubescence and shape of the pod details were presented in Table 1. The leaf shape variability was noted to be lanceolate, pointed ovate and round ovate (Figure 1a). The genotypes had pointed ovate leaf shapes, with the exception of genotypes GM-2, GM-7, GM-4, GM-12, GM-14, GM-15, GM-19 and GM-20, which had lanceolate leaf shapes, and genotypes GM-11, GM-16, GM-13, GM-18, GM-24, GM-22 and GM-25, which had circular ovate leaf shapes. The leaf color showed noticeable variation from normal to dark green, all the genotypes were green leaf color, except the genotypes GM-2, GM-4, GM-15, GM-22, GM-8, GM-13, GM-11, GM-25 and GM-26 (Table 1). The flower colors (Figure 1c) of genotypes had white, except GM-3, GM-8, GM-6 and GM-13, which have purple flowers. The genotypes were noted for distinct growth habit of determinate, indeterminate and semi-determinate. Semi-determinate types were distinguished from indeterminate and determinate types, which were with highly expressed SAMs than indeterminate lines, since vegetative operation of shoot apical meristems (SAM) ceases soon after inflorescence initiation in determinate types of soybean, whereas in indeterminate types the terminal bud continues with SAM during the growing season, semi-determinate types were distinguished from indeterminate and determinate types, which were with highly expressed SAMs [12,13,14] (Figure 1b). The pod pubescence was absent in all the genotypes except in the genotypes GM-11, GM-16, GM-19 and GM-25. The genotypes (Table 1) had pods that varied in color from dark to light green, with flat, slightly curved and curved pod forms.

Genetic variability is the key factor for plant breeding, the magnitude of heterogeneity in germplasm might be influenced by geneticvariation (GV) and phenotypicvariation (PV) of traits. Estimates of genotypic and phenotypic variance (GCV and PCV) of agronomical and biochemical traits (Table 2) showed a wide genetic base for 14 traits out of 18 characters, which is useful for genotype selection including its trait of interest.

The results revealed that PCV has a higher value than GCV in all traits, with varying degrees of phenotypic variance due to genetic variance within the population for all parameters (18 traits), implying that GCV and PCV could reveal the extent of population variability. Furthermore, to facilitate the correlation of genetic effects to advance the selection, the estimation of heritability came in force, and in order to establish the PV within populations due to heritable or non-heritable genetic effects, the assessment of heritability was implemented. The results revealed that the enhanced heritability estimation values of unique individual characteristics within the population (Table 2) showed that these characters can be used to forecast the selection process.

The 18 characteristics of population genotypes indicate that phenotypic variability is greater than the genotypic variability, and increased heritability associated with increased genetic advance than the percentage mean (Table 2) of most of the traits. It could be concluded that additive gene action influenced these traits, hence selection is most effective.

**Figure 2:**
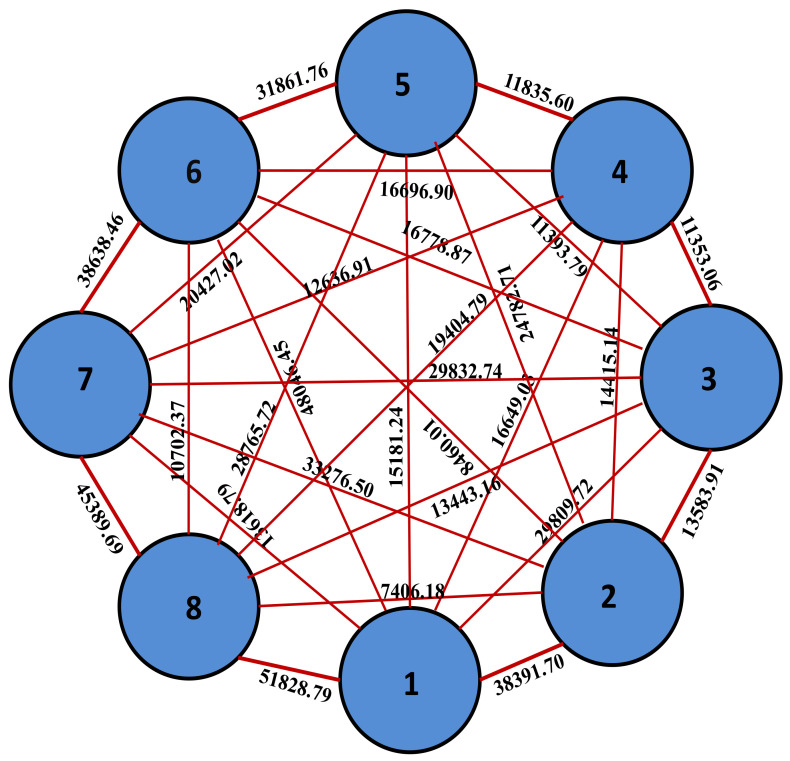
Divergent groups of vegetable soybean genotypes were grouped into cluster I (1) to cluster VIII (8) segregated by cluster distances indicating that cluster I and cluster-VIII depicted maximum genetic divergence.

The vegetable soybean divergence was determined using data from 18 traits of 28 genotypes, and the clusters divided using Trocher’s method [15]. The estimated D^2^ values as the squares of generalized intervals, the genotypes were divided into eight clusters (Figure 2), and Table 3 represents the genotype cluster distribution pattern. Cluster II incorporated large number (8) of genotypes and was followed by cluster V and cluster I, each including four genotypes; cluster III and IV contained three genotypes and each of the remaining clusters VI, VII and VIII included only two genotypes. The maximum genetic distance was found between cluster VIII and I (D^2^ = 51,828.79) followed by cluster VI and I. The genotypes GM-6 and GM-27 from cluster VIII and GM-10, GM-18, GM-20 and GM-25 from cluster I had the greatest genetic distance.

## 3. Discussion

The morphological characteristics of vegetable soybean genotypes were determined using soybean descriptors for leaf structure, leaf color, flower color, pod color, pubescence and shape. These characteristics can assist breeders in genotype selection dependent on phenotype, as well as in genetic improvement projects. In the vegetable soybean genotypes type’s, the semi-determinate growth habit types have an immense potential in for pod yield, this could be due to the shorter stem length than indeterminate types (prone to lodging), thus lead to more number nodes with inflorescence, while determinate types abruptly terminate their growth after flowering [13,14]. The results depict the presence of sufficient morphological traits variability between the genotypes. Based on these morphological traits, it could be possible to identify elite genotypes for vegetable purposes within the genotype population; in the same sense, differences in genetic variation were found between soybean genotypes [16,17,18].

Among18 characteristics,14 traits were confirmed the broad genetic base through high GCV, and PCV estimates (Table 2), and therefore these traits useful for selection of genotypes in the further vegetable soybean breeding. Similarly, significant and maximum variability as GCV, PCV was reported for plant height and pod yield per plant [8,18,19,20,21,22,23,24]. The traits like days to vegetable pod maturity, length and width of pod, number of harvests, starch content were found to be moderate GCV, and PCV (Table 2), showing narrow genetic base among the genotypes, and these findings inferred that less scope for genotypic selection using these traits, and these results were underlined with previous studies of earliness traits [16,22,25,26,27].

The parameters (18 traits) depicted high variability (PCV, GCV) coupled with high heritability (*h*^2^) (Table 2) and indicated the influence of additive gene action on these traits; hence selection could be most effective. Similarly, the findings showed that pod number and plant height have a high heritability [16,28,29], days to 50% flowering and height [17,30], plant height and pods number and length [10], days to vegetable maturity, height and pods number [18], pods number [31,32], pod length [33] and earliness to flowering and vegetable maturity [27].

The variables (18traits) represented high variability (PCV, GCV); heritability(*h*^2^) coupled with genetic advance over percentage mean (GAM) (Table 2) revealed these traits were showed additive gene action. The selection of the genotypes based on these traits is most effective for vegetable soybean improvement, since traits governed by additive gene action. In soybeans, inline findings were obtained for plant height and pod number [28,29], plant height, early flowering, maturity and pod number traitshave also been considered [17,27,30,31,32], along with pod length [33].

Eight clusters were found by screening all genotypes based on estimated D^2^ values as the squares of generalized distances (Figure 2). The distance between the clusters VIII and I (D^2^ = 51828.79) was the maximum and was followed by the distance between the clusters VI and I (D^2^ = 48046.45). The maximum genetic distance was exhibited by the genotypes GM-6 and GM-27 from the cluster VIII, and GM-10, GM-18, GM-20 and GM-25 in the cluster Iin terms of the genetic divergence. The genotypes GM-6 and GM-27 were superior for yield traits. Therefore, the high yielding divergent genotypes can be used for breeding yielding varieties, by crossing and transfer of yield traits into the low yielding genotypes of divergent clusters [10,34,35]. The divergent parents were very important for further crop improvement, it assists in the implementation of the best breeding technique that uses additive and non-additive traits to produce the best genetically enhanced vegetable soybean.

## 4. Materials and Methods

### 4.1. Experimental Details

All field experiments were accomplished at the experimental field at the Department of Vegetable Science, College of Horticulture, Vellanikkara, Kerala Agricultural University, Kerala, India during October to December 2017. The experimental site situated at an altitude of 23 m above sea level, between 10° 32” N latitude and 76° 16” E longitude. The field experiment was conducted for the evaluation and assessment of agronomical and biochemical traits. The design used was randomized blocks design that contained three replications. The genotypes namely GM-1 to GM-28 (Table 1) was provided by Indian Council of Agricultural Research, Indian Institute of Horticultural Research (ICAR-IIHR), Bengaluru, Karnataka, India for research purpose. Seeds were sown at a spacing of 45 cm × 45 cm apart, in each treatment 20 plants pergenotype were grown. The guidelines regarding plant production and agronomical activities were provided by the Kerala Agricultural University [36]. Random five plants were selected from each treatment to record the observations of parameters.

### 4.2. Biochemical Analysis

Fresh pods of vegetable soybean are used for the estimation of biochemical trait analysis. The anthrone reagent procedure was used to estimate carbohydrate and starch content, wherein carbohydrates were dehydrated by concentrated H_2_SO_4_ to form furfural. The reagent’s active form is anthranol, the enol tautomer of anthrone, which interacts with the carbohydrate furfural derivative to produce a green color in dilute solutions and a blue color in concentrated solutions, which was calculated calorimetrically at A_620_nm [37]. Lowry’s procedure was used to assess the protein content, in which oxidation of aromatic amino acids is catalyzed by alkaline CuSo_4_, which is preceded by the reduction of sodium potassium molybdate tungstate of Folin’s reagent, resulting in a purple-colored complex. The color intensity was considered to be equivalent to the amount of aromatic amino acids in the sample solution [38]. Vitamin C in pods was estimated by using 2, 6 di-chloro indo-phenol dye method, the 2, 6-dichlorophenol indophenol dye is reduced to a colorless leuco-base by ascorbic acid. Dehydroascorbic acid is formed when ascorbic acid is oxidized. Despite the fact that the dye was a blue-colored chemical, finally resulted into pink color, in an acidic medium (oxalic acid) titrating medium [39]. Polyphenols estimation was carried out with Folin-Ciocalteau reagent method [40]. Phosphorus content in the fresh pods was estimated by Vanado-molybdo-phosphoric acid (Barton’s reagent) reagent method. The yellow color solvent was subjected for absorbance at a wavelength of A_470_ nm. The intensity of the yellow color from this complex formation was proportional to the phosphate concentration [41], calcium and iron content in the fresh pods were estimated by ICP-AES [42].

### 4.3. Statistical Analysis

The agronomical and biochemical traits were averaged and the mean data were entered into the statistical program IndoStat Version 9.3, the results were interpreted as described by [43].The genotype variance is combined with the environmental variance to form phenotypic variance (PV), and the additive genetic variation, dominance variance, and epistatic variance are the three main components of genotypic variance (GV), these variances were estimated among the vegetable soybean genotype to reveal the genetics of genotypes [44] in collaboration with colleagues from King Saud and Princess Nourah bint Abdulrahman Universities.

(1) $$\mathrm{Genotypic}\;\mathrm{variance}\;(\mathrm{GV})\;=\;\frac{\mathrm{Genotype}\;\mathrm{mean}\;\mathrm{square}\;\left(\mathrm{gms}\right)-\mathrm{Error}\;\mathrm{mean}\;\mathrm{square}(\mathrm{ems})}{\mathrm{Number}\;\mathrm{of}\;\mathrm{replication}\;(r)}$$

(2) $$\mathrm{Phenotypic}\;\mathrm{variance}\;\left(\mathrm{PV}\right)\;=\;\mathrm{GV}+\mathrm{EV}/r$$

Genotypic coefficient of variation (GCV), phenotypic coefficient of variation(PCV) were classified as high (>20%), moderate (10–20%) and low (0–10%) [45] and were calculated with the following formulas:

(3) $$\mathrm{Genotypic}\;\mathrm{coefficient}\;\mathrm{of}\;\mathrm{variation},\;\mathrm{GCV}\;(\%)\;=\;\sqrt{\frac{\mathrm{GV}}{\bar{x}}}\times 100$$

(4) $$\mathrm{Phenotypic}\;\mathrm{coefficient}\;\mathrm{of}\;\mathrm{variation},\;\mathrm{PCV}\;(\%)\;=\;\sqrt{\frac{\mathrm{PV}}{\bar{x}}}\times 100$$

The heritability (*h*^2^) is account for a proportion of trait variation of both genetic factors that is dominance and gene-gene interactions, percentage was categorized as high (>60%), moderate (31–60%) and low (0–30%) [46,47], which was estimated as follows:

(5) $$\mathrm{Heritability},\;h^{2}\;=\;\frac{\mathrm{GV}}{\mathrm{PV}}$$

Genetic advance and genetic advance mean reveal the direct relationship between heritability and response to selection, which were classified as high (>20%), moderate (11–20%) and low (0–10%) [48], and was calculated as follows:

(6) $$\mathrm{Genetic}\;\mathrm{advance},\;\mathrm{GA}\;=\;K\sqrt{\mathrm{PV}\;\times \;h^{2}}$$

(7) $$\mathrm{Genetic}\;\mathrm{advance}\;\mathrm{mean},\;\mathrm{GAM}\;=\;\frac{\mathrm{GA}}{\bar{x}}$$

where,

$\bar{x}$ = standard error of the mean

PV = phenotypic variance

*h*^2^ = broad sense heritability

K = selection intensity differential

## 5. Conclusions

The findings of the research revealed a high prevalence of genetic heterogeneity among genotypes, both genotypic and phenotypic variation. For agronomical and biochemical traits with broad genetic base, high GCV and PCV were found, which could be useful in genotype selection in subsequent generations of vegetable soybean breeding. Additive gene action provides a basis for high heritability estimates and genetic advance over percent mean of traits, and hence selection will be more effective in improvement of vegetable soybean. Due to the high genetic variation among genotypes, the genotypes GM-6 and GM-27 were substantially more divergent from the genotypes GM-10, GM-18, GM-20 and GM-25 (cluster I) than the other genotypes. The genotypes GM-6 and GM-27 (cluster VIII) were agronomically superior in yield attributing traits. Hence, these genotypes could be used for commercial cultivation and in genetic improvement programs. Furthermore, they could be used in diverse parental crosses, which are likely to create heterotic hybrids, to help the production of even more strongly diverse lines.

## Figures and Tables

**Table 1:** The morphological characterization of of vegetable soybean genotypes using soyabean descriptor (IBPGR, 1984).

Genotypes	Leaf Shape	Leaf Color	Flower Color	Growth Habit	Pod Color	Pod Pubescence	Pod Shape
GM-1	Pointed ovate	Green	Purple	Determinate	Green	Absent	Slightly curved
GM-2	Lanceolate	Dark green	Purple	Semi determinate	Green	Absent	Straight
GM-3	Pointed ovate	Green	White	Semi determinate	Dark green	Absent	Straight
GM-4	Lanceolate	Dark green	Purple	Determinate	Green	Absent	Slightly curved
GM-5	Pointed ovate	Green	Purple	Semi determinate	Green	Absent	Curved
GM-6	Pointed ovate	Green	White	Semi determinate	Green	Absent	Slightly curved
GM-7	Lanceolate	Green	Purple	Indeterminate	Green	Absent	Slightly curved
GM-8	Pointed ovate	Dark green	White	Determinate	Dark green	Absent	Slightly curved
GM-9	Pointed ovate	Green	Purple	Indeterminate	Green	Absent	Slightly curved
GM-10	Pointed ovate	Green	Purple	Determinate	Green	Absent	Slightly curved
GM-11	Round ovate	Dark green	Purple	Determinate	Green	Present	Slightly curved
GM-12	Lanceolate	Green	Purple	Indeterminate	Green	Absent	Curved
GM-13	Round ovate	Dark green	White	Determinate	Green	Absent	Straight
GM-14	Lanceolate	Green	Purple	Determinate	Green	Absent	Straight
GM-15	Lanceolate	Dark green	Purple	Semi determinate	Green	Absent	Slightly curved
GM-16	Round ovate	Green	Purple	Determinate	Green	Present	Straight
GM-17	Pointed ovate	Green	Purple	Indeterminate	Green	Absent	Curved
GM-18	Round ovate	Green	Purple	Determinate	Green	Absent	Slightly curved
GM-19	Lanceolate	Green	Purple	Semi determinate	Green	Present	Curved
GM-20	Lanceolate	Green	Purple	Semi determinate	Green	Absent	Straight
GM-21	Pointed ovate	Green	Purple	Indeterminate	Green	Absent	Slightly curved
GM-22	Round ovate	Dark green	Purple	Determinate	Dark green	Absent	Slightly curved
GM-23	Pointed ovate	Green	Purple	Semi determinate	Green	Absent	Slightly curved
GM-24	Round ovate	Green	Purple	Determinate	Green	Absent	Straight
GM-25	Round ovate	Dark green	Purple	Determinate	Green	Present	Slightly curved
GM-26	Pointed ovate	Dark Green	Purple	Indeterminate	Green	Absent	Curved
GM-27	Pointed ovate	Green	Purple	Indeterminate	Green	Present	Slightly curved
GM-28	Pointed ovate	Green	Purple	Indeterminate	Green	Absent	Curved

**Table 2:** Estimates of components of genetic advance, heritability, variance and genetic advance over percentage of mean for morphological and quality parameters in vegetable soybean.

Sl. No.	Character	GV	PV	GCV (%)	PCV (%)	*h*^2^ (%)	GA (%)	GAM (%)
1.	Plantheight (cm)	392.73	394.78	43.21	43.33	99.46	40.71	88.77
2.	Daysto50% flowering	12.89	14.20	10.51	11.05	90.47	7.03	20.59
3	Daystofirst harvest	19.87	22.93	9.79	10.54	86.23	8.52	18.73
4	Daytovegetable maturity	2.90	3.37	13.41	14.49	85.58	3.24	25.55
5	Podlength (cm)	0.36	0.36	12.88	12.91	99.64	1.23	26.50
6	Podwidth (cm)	0.09	0.09	14.49	14.56	99.11	0.64	29.73
7	Pod yieldperplant (g)	361.27	458.52	29.94	33.87	78.11	34.55	54.51
8	Podsperplant	181.16	191.61	42.27	43.51	94.35	26.92	84.58
9	Podweight (g)	0.52	0.56	29.40	30.67	91.92	1.42	58.08
10	Numberofharvests	0.71	0.86	19.64	20.41	92.56	1.76	38.93
11	Starch (g/100 g)	0.29	0.29	35.54	34.66	99.30	70.91	70.91
12	Carbohydrate (g/100 g)	3.84	3.85	23.99	24.01	99.87	49.39	49.39
13	Protein (g/100 g)	10.52	10.57	23.61	23.67	99.51	48.53	48.53
14	Vitamin C (mg/100 g)	8.06	8.09	33.21	33.27	99.66	68.30	68.30
15	Iron (mg/100 g)	6.29	6.30	50.19	50.24	99.80	100.00	103.29
16	Calcium (mg/100 g)	90.12	90.14	39.53	39.54	99.97	81.43	81.426
17	Phosphorous (mg/100 g)	13060.89	13205.34	24.87	25.01	98.86	50.94	50.94
18	Polyphenols (g/100 g)	7.71	7.84	43.32	43.45	99.38	88.97	88.97

GV—genotypic variation, PV—phenotypic variation, GCV—genotypic coefficient of variation, PCV—phenotypic coefficient of variation, *h*^2^—heritability, GA—genetic advance, GAM—genetic advance over percentage of mean.

**Table 3:** Cluster composition based on D^2^ statistics in vegetable soybean.

Cluster	Genotypes Per Cluster	Names of the Genotypes
I	4	GM-10, GM-18, GM-20, GM-25
II	8	GM-2, GM-3, GM-12, GM-8, GM-9, GM-13, GM-28, GM-14
III	3	GM-7, GM-21, GM-15
IV	3	GM-5, GM-19, GM-11
V	4	GM-24, GM-22, GM-26, GM-23
VI	2	GM-1, GM4
VII	2	GM-16, GM-24
VIII	2	GM-6, GM-27

## Data Availability

All data are available within this publication.

## References

[1] Shanmugasundaram S., Yan M.R. Vegetable soybeans for nutritional quality income generation and soil sustainability. Proceedings of the World Soybean Research Conference VI.

[2] Mebrahtu A.T., Devine T.E. (2010). Combining ability analysis for selected green pod yield components of vegetable soybean genotypes (*Glycine max* L.). N. Z. J. Crop Hort. Sci..

[3] Keatinge J.D.H., Easdown W.J., Yang R.Y., Chadha M.L., Shanmugasundaram S. (2011). Overcoming chronic malnutrition in a future warming world: The key importance ofmungbean and vegetable soybean. Euphytica.

[4] Fehr W.R., Caviness C.E. (1977). Stages of soybean development. Iowa State University Cooperative Extension Service, Special Report.

[5] Masuda R. Quality requirement and improvement of vegetable soybean.Vegetable Soybean: Research Needs for Production and Quality Improvement. Proceedings of the a Workshop.

[6] Mathur S. (2004). Soybean wonder legume. Beverage Food World.

[7] Shanmugasundaram S., Yan M.R. Global expansion of high value vegetable soybean. Proceedings of the 7th World Soybean Research Conference.

[8] Mishra S., Pancheshwar D.K., Singh P., Jha A. (2014). study of genetic variability in recently evolved genotypes of soybean (*Glycine max* (L.) Merill). Trends Biosci..

[9] Baraskar V.V., Kacchadia H.V., Vacchan J.H., Barad H.R., Patel M.B., Darwankar M.S. (2014). Genetic variability, heritability and genetic advance in soybean [*Glycine max* (L.) Merrill]. Electron. J. Plant Breed..

[10] Kumar A., Pandey A., Aochen C., Pattanayak A. (2015). Evaluation of genetic diversity and interrelationships of agromorphological characters in soybean (*Glycine max* L.) genotypes. Proc. Natl. Acad. Sci. India B.

[11] IBPGR (1984). Disriptors for Soybean. International Board for Plant Genetic Resources.

[12] Boerma H.R., Specht J.E., Carlson J.B., Lersten N.R., Boerma H.R., Specht J.E. (1987). Reproductive morphology. Soybeans: Improvement, Production, and Uses.

[13] Lockhart J., Candat A., Paszkiewicz G., Neveu M., Gautier R., Logan D.C., Avelange-Macherel M.-H., Macherel D. (2014). Finding Dt2, the Dominant Gene That Specifies the Semideterminate Growth Habit in Soybean. Plant Cell.

[14] Kato S., Sayama T., Taguchi-Shiobara F., Kikuchi A., Ishimoto M., Cober E. (2019). Effect of change from a determinate to a semi-determinate growth habit on the yield and lodging resistance of soybeans in the northeast region of Japan. Breed. Sci..

[15] Rao C.R. (1952). Advanced Statistical Methods in Biometrical Research.

[16] Basavaraja G.T., Naidu G.K., Salimath P.M. (2005). Evaluation of vegetable soybean genotypes for yield and component traits. Kar. J. Agric. Sci..

[17] Reni Y.P., Raob Y.K. (2013). Genetic variability in soybean (*Glycine max* (L.) Merrill). Inter. J. Plant Anim. Environ. Sci..

[18] Pagde L., Abubakkar D., Ingole G., Dhuppe M.V. (2015). Study of genetic variability for yield and yield contributing traits in soybean (*Glycine max* (L.) Merrill). Bioinfolet.

[19] Mebrahtu T., Mohamed A. (2006). Genetic variation for green pod yield and quality among vegetable soybean genotypes. J. Crop Improv..

[20] Poornima R., Koti R.V., Nair R.N. (2014). Physiological basis of yield variation in vegetable soybean and organoleptic test for acceptance. Plant Arch..

[21] Ramya V., Mummigatti U.V. (2015). Characterization of vegetable soybean genotypes for phenological, physiological and yield attributing traits. Kar. J. Agric. Sci..

[22] Haruna M.K., Turaki Z.G.S., Bibinu A.T.S., Wali A.S. (2015). Soybean varietal evaluation in Northern Guinea Savanna. J. Bio. Agric. Healthcare.

[23] Sureshrao S.S., Singh V.J., Gampala S., Rangare N.R. (2014). Assessment of genetic variability of the main yield related characters in soybean. Int. J. Food Agric. Veterin. Sci..

[24] Mahbub M.M., Rahman M.M., Hossain S., Mahmud F., Mir M.M. (2015). Genetic variability, correlation and path analysis for yield and yield components in soybean. Am.-Eur. J. Agric. Environ. Sci..

[25] Sharma B.K., Kushwah S.S., Verma K.S., Singh O.P. (2013). Studies on french bean (*Phaseolus vulgaris* L.) varieties under different N, P, K and S levels for growth, yield and economics. J. Hort. Sci..

[26] Njoroge J.N., Owouche J.O., Oyoo M.E. (2015). Evaluation of soybean (*Glycine max* (L.) Merrill) genotypes for agronomic and quality traits in Kenya. Afr. J. Agric. Res..

[27] Kuswantoro H. (2017). Genetic variability and heritability of acid-adaptive soybean promising lines. Biodiversitas.

[28] Gohil V.N., Pandya H.M., Mehta D.R. (2006). Genetic variability for seed yield and its component traits in soybean. Agric. Sci. Digest..

[29] Karnwal M.K., Singh K. (2009). Studies on genetic variability, character association and path coefficient for seed yield and its contributing traits in soybean (*Glycine max* (L.) Merrill). Legume Res..

[30] Dilnesaw Z., Abadi S., Getahun A. (2013). Genetic variability and heritability of soybean (*Glycine max* (L.) Merrill) genotypes in Pawe district, Metekelzone, Benishangule-Gumuz regional state, north western Ethiopia. WudpeckerJ. Agric. Res..

[31] Ekka P.K., Lal G.M. (2016). Study on genetic variability and character association in soybean (*Glycine max* (L.) Merrill) germplasm at Vindhyan zone of Uttar Pradesh. Agric. Sci. Digest..

[32] Manav A.R.N. (2017). Genetic variability studies for yield and seedling traits in soybean (*Glycine max* (L.) Merrill). Indian Res. J. Genetic Biotech..

[33] Thakur D.K., Gendley T.K., Tigga K., Sharma A.C. (2015). Study on genetic variability, heritability and genetic advance for seed yield and its attributing traits in soybean [*Glycine max* (L.) Merrill]. Trends Biosci..

[34] Sood V.K., Sood V.P., Pathania A., Chandel K. (2006). Exploiting genotypic variability in relation to genetic divergence among advanced lines of soybean (*Glycine max* (L) Merrill). Indian J. Plant Genet. Res..

[35] Patil S.S., Naik M.R., Patil A.B., Ghodke U.R. (2011). Genetic diversity in soybean. Legume Res..

[36] KAU (2016). Package of Practices Recommendation: Crops.

[37] Yemm E.W., Willis A.J. (1954). The estimation of carbohydrates in plant extracts by anthrone. Biochem. J..

[38] Lowry O.H., Rosebrough N.J., Farr A.L., Randall R.J. (1951). Protein measurement with the folin-phenol reagent. J. Biol. Chem..

[39] Sadasivam S., Balasubraminan T. (1987). Practical Manual in Biochemistry.

[40] Sadasivam S., Manickam A. (1992). Biochemical Methods for Agricultural Sciences.

[41] Jackson M.L. (1973). Soil Chemical Analysis.

[42] Piper C.S. (1966). Soil and Plant Analysis.

[43] Panse V.G., Sukhatme P.V. (1967). Statistical Methods for Agricultural Workers.

[44] Wricke G., Weber W.E. (1986). Quantitative Genetics and Selection in Plant Breeding.

[45] Shivasubramanian S., Menon N. (1973). Heterosis and inbreeding depression in rice. Madras Agric. J..

[46] Robinson H.F., Comstock R.E., Harvey V.H. (1949). Estimates of heritability and degree of dominance in corn. Agron. J..

[47] Burton C.W., Devane E.H. (1953). Estimating heritability in tall Fescue (*Festuca arundinaceae*) from replicated clonal material. Agron. J..

[48] Johnson H.W., Robinson H.F., Comstock R.E. (1955). Estimates of genetic and environmental variability in soybeans. Agron. J..

